# The CONDUCT‐AF trial: Rationale and design of a prospective, randomized, multicentre study comparing conduction system and biventricular pacing in patients undergoing atrioventricular node ablation for heart failure with atrial fibrillation

**DOI:** 10.1002/ejhf.70013

**Published:** 2025-09-08

**Authors:** Maja Ivanovski, Miha Mrak, Anja Zupan Mežnar, Matevž Jan, Catalin Pestrea, Sandro Brusich, Peter Bogyi, Sebastiaan Dhont, Zrinka Jurišić, Vassil Traykov, Borka Pezo Nikolić, Wilfried Mullens, David Žižek

**Affiliations:** ^1^ Cardiology Department University Medical Centre Ljubljana Ljubljana Slovenia; ^2^ University of Ljubljana, Faculty of Medicine Ljubljana Slovenia; ^3^ Department of Cardiovascular Surgery University Medical Centre Ljubljana Ljubljana Slovenia; ^4^ Department of Interventional Cardiology Brasov County Emergency Clinical Hospital Brasov Romania; ^5^ Clinic for Cardiovascular Diseases, Clinical Hospital Centre Rijeka Rijeka Croatia; ^6^ University of Rijeka Department of Internal Medicine, Medical Faculty Rijeka Croatia; ^7^ Central‐Hospital of Northern Pest ‐ Military Hospital Budapest Hungary; ^8^ Semmelweis University, Centre for Translational Medicine Budapest Hungary; ^9^ Department of Cardiology Ziekenhuis Oost‐Limburg A.V. Genk Belgium; ^10^ Hasselt University, Faculty of Medicine and Life Sciences Diepenbeek Belgium; ^11^ Department of Cardiology University Hospital Center Split Split Croatia; ^12^ Department of Invasive Electrophysiology Clinic of Cardiology, Acibadem City Clinic Tokuda University Hospital Sofia Bulgaria; ^13^ University Hospital Centre Zagreb Zagreb Croatia

**Keywords:** Atrioventricular node ablation, Atrial fibrillation, Conduction system pacing, Biventricular pacing, Heart failure

## Abstract

**Aims:**

There is a lack of data from randomized clinical trials comparing treatment outcomes between conduction system pacing (CSP) modalities and biventricular pacing (BVP) in symptomatic patients with refractory atrial fibrillation (AF) scheduled for atrioventricular node ablation (AVNA). The CONDUCT‐AF investigates whether CSP is non‐inferior to BVP in improving left ventricular ejection fraction (LVEF) and clinical outcomes in heart failure (HF) patients with symptomatic AF undergoing AVNA.

**Methods:**

This study is an investigator‐initiated, prospective, randomized, multicentre clinical trial conducted across 10 European centres, enrolling 82 patients with symptomatic AF, HF with reduced LVEF, and narrow QRS. Participants will be randomized 1:1 to CSP or BVP with subsequent AVNA and followed for at least 24 months. The primary endpoint is the change in LVEF after 6 months. Secondary endpoints will include time to the first occurrence of worsening HF or cardiovascular death and its individual components, total number of HF hospitalizations, change in quality of life, N‐terminal pro‐B‐type natriuretic peptide, 6‐min walk test distance, and safety outcomes.

**Conclusions:**

The CONDUCT‐AF trial will provide critical insights into the optimal pacing modality for patients with HF and refractory AF undergoing AVNA. Recruitment is expected to conclude in 2025, with the first study results anticipated in 2026.

## Introduction

### Atrial fibrillation in heart failure

Disorganized atrial activity during atrial fibrillation (AF) has many deleterious haemodynamic effects contributing to cardiac remodelling and induction of fibrosis.^1^, ^2^, ^3^ Early studies proved that irregularity and loss of atrial contribution to left ventricular (LV) filling decreased cardiac output (CO).^4^ Further exploration revealed that independent of rate, irregular ventricular rhythm disables cardiac contractility to adapt during beat‐to‐beat changes in ventricular filling. In addition, AF leads to insufficient coronary flow, molecular LV remodelling, and disrupted Ca^2+^ homeostasis, which eventually may induce contractile dysfunction.^3^, ^5^ In brief, deleterious haemodynamic effects of AF on cardiac function can be attributed to loss of atrial contraction, reduced CO, and irregular and often rapid heart rate.^2^, ^6^ Considering these facts, removing irregularity and fast ventricular response alone can increase CO and prevent long‐term deleterious effects of AF culminating in arrhythmia‐induced cardiomyopathy.

### Treatment strategies

Catheter ablation enables restoration of sinus rhythm and several studies have recently demonstrated its prognostic benefit.^7^, ^8^, ^9^ Therefore, current guidelines recommend CA as a first‐line treatment when arrhythmia‐induced cardiomyopathy is highly probable (class I, level of evidence B, European and US guidelines).^10^, ^11^ However, these benefits might be less distinct in patients with long‐standing, persistent AF in whom lower interventional success rates might be expected.^12^ Additionally, most trials do not specify ablation outcomes in persistent AF or elderly patients with more comorbidities in whom recurrences may be particularly problematic. Therefore, even though the guidelines recommend CA as a treatment of choice, alternative therapeutic strategies may be considered. Atrioventricular node ablation (AVNA) with permanent pacemaker implantation (pace and ablate strategy) is a feasible rate control option that offers more robust control of ventricular rate over medical therapy alone, as well as definitive rhythm regularization.^13^ A recent meta‐analysis compared the efficacy and safety endpoints among five major approaches for AF. Although CA performed best in reducing AF recurrence, the pace and ablate strategy showed superiority in reducing mortality and rehospitalization.^14^ Current 2024 European Society of Cardiology guidelines recommend AVNA in patients unresponsive or intolerant to intensive rate and rhythm control therapy, and not eligible for rhythm control by CA (class IIa, level of evidence B). The choice of pacing modality depends on patient characteristics and the presence of heart failure (HF).^10^

### Pacing modalities in the pace and ablate strategy

Right ventricular pacing (RVP) combined with AVNA has demonstrated its potential to effectively improve clinical outcomes.^15^ However, the beneficial effects of rate control and regularization after AVNA could be hampered by non‐physiological asynchronous contraction after RVP.^16^ Deleterious effects of RVP can be avoided with biventricular pacing (BVP). As demonstrated in the APAF‐CRT study, BVP combined with AVNA significantly reduced mortality, HF hospitalizations, and improved LV function.^17^, ^18^ However, the benefit was less distinct in symptomatic AF patients with moderately reduced left ventricular ejection fraction (LVEF) and narrow QRS. BVP circumvents the dyssynchrony induced by RVP but still induces abnormal epicardial activation that results in QRS prolongation and some extent of electrical and mechanical dyssynchrony.^3^, ^17^, ^19^

In recent years, conduction system pacing (CSP) modalities, including His bundle pacing (HBP) and left bundle branch area pacing (LBBAP), have evolved as a promising alternative allowing the most physiological activation of the myocardium. Retrospective studies demonstrated both the feasibility and advantages of HBP in echocardiographic outcomes and time to death or HF hospitalization compared to conventional RVP.^20^ Although the His bundle appears to be the ideal physiological pacing site, its proven feasibility alongside AVNA must be considered in light of some significant limitations. The implant technique is challenging and requires advanced implantation tools and longer procedural and fluoroscopic times. Low sensing values could engender atrial oversensing in pacemaker‐dependent patients. Moreover, higher pacing thresholds and their further rise after AVNA due to the proximity of the ablation site to the implanted HBP lead might be observed, necessitating a right ventricular (RV) backup lead and early battery replacement.^21^, ^22^ In contrast, LBBAP lead is implanted in the region of the left bundle in the septum, which has an adequate distance from the AVNA site. Therefore, this modality could minimize the risk of an increase in capture threshold after AVNA. Additionally, the pacing parameters of LBBAP were stable in long‐term follow‐up studies, precluding the need for back‐up pacing and early battery replacement. Therefore, compared to HBP, LBBAP may offer a more feasible physiological pacing option, especially in patients undergoing AVNA.^21^

Some observational studies have already shown superior symptomatic and echocardiographic improvement in symptomatic AF patients who underwent CSP, both HBP and LBBAP, compared to BVP after AVNA, especially in HF patients with narrow baseline QRS and reduced LVEF (<50%).^23^, ^24^ Therefore, it is reasonable to assume that CSP, in conjunction with AVNA, could represent a promising alternative to BVP in refractory AF patients. Nevertheless, the 2021 guidelines give BVP a class IIa recommendation for those with mildly reduced and a class I for those with reduced LVEF. In contrast, HBP is allowed as an alternative with class IIb, and recommendations for LBBAP remain to be formulated.^25^

### Study rationale and aim

Data from randomized clinical trials comparing treatment outcomes between CSP and BVP in combination with AVNA are lacking. The need for a comparison between both pacing modalities in the treatment of refractory symptomatic AF constituted the rationale for the CONDUCT‐AF study.

## Study design

CONDUCT‐AF is an investigator‐initiated, prospective, multicentre, randomized, single‐blinded, two‐arm clinical trial conducted in 10 European centres (ClinicalTrials.gov: NCT05467163). Eighty‐two patients with symptomatic AF, HF, and narrow QRS will be randomized in a 1:1 ratio to CSP or BVP with subsequent AVNA (*Graphical Abstract*) using a computer‐generated randomization sequence stratified by centre, gender, and LVEF below or above 35%.

The study will follow the Declaration of Helsinki, Good Clinical Practice, and the applicable local regulatory requirements. It was initiated after approval from the institutional review and ethics board at each centre. The authors, who are responsible for writing this paper and its final content, designed the protocol.

### Study population and enrolment

Patients with reduced LVEF (<50%), narrow intrinsic QRS (≤120 ms), initial N‐terminal pro‐B‐type natriuretic peptide (NT‐proBNP) >600 ng/L, and refractory symptomatic AF despite guideline‐directed medical therapy or failed catheter ablation are candidates for participation in this study. Detailed inclusion and exclusion criteria are presented in *Table* *1*. All patients will receive detailed clarification from local investigators and will provide their written informed consent before enrolment. A screening log with reasons for inclusion failure will be collected at each site.

**Table 1:** Inclusion and exclusion criteria in the study

Inclusion criteria	Exclusion criteria
Symptomatic permanent AF refractory to drug therapy or failed catheter ablation Left ventricular ejection fraction <50% Narrow intrinsic QRS ≤120 ms NT‐proBNP >600 ng/L Age between 18 and 85 years	Pre‐existing permanent PM, ICD or CRT device with >5% of paced beats (i.e. backup pacing) Life expectancy <12 months Severe concomitant non‐cardiac disease Pregnancy Recent (<3 months) MI, percutaneous or surgical myocardial revascularization Significant heart valve disease (severe insufficiency or stenosis) Contraindication for oral anticoagulation Mechanical tricuspid valve replacement Unwillingness to participate or lack of availability for follow‐up

AF, atrial fibrillation; CRT, cardiac resynchronization therapy; ICD, implantable cardioverter‐defibrillator; MI, myocardial infarction; NT‐proBNP, N‐terminal pro‐B‐type natriuretic peptide; PM, pacemaker.

Eligible patients will undergo baseline evaluation including clinical history, 12‐lead electrocardiogram, assessment of New York Heart Association (NYHA) class and European Heart Rhythm Association score of AF (EHRA‐AF), echocardiographic examination, assessment of quality of life, 6‐min walk test (6MWT), and blood testing. Quality of life and symptoms will be evaluated with the Kansas City Cardiomyopathy Questionnaire (KCCQ). Subjects fulfilling all inclusion criteria, without meeting any exclusion criteria, will be automatically randomized in a 1:1 ratio to BVP + AVNA or CSP + AVNA. Randomization will be performed automatically according to the previously generated random allocation list. Both groups will be treated identically in all respects except for the intervention being tested. There will be no restrictions on medical therapy adjustments during the study. Optimization of guideline‐directed medical therapy following HF guidelines will be encouraged, but at the discretion of the treating physician.

### Study objectives and endpoints

The CONDUCT‐AF study aims to confirm the non‐inferiority of CSP versus BVP in echocardiographic and clinical outcomes, during a 24‐month follow‐up in HF patients with symptomatic AF and narrow QRS scheduled for AVNA. The study primary endpoint is the LVEF change from baseline to 6 months. Changes in indexed LV end‐diastolic and end‐systolic volumes from baseline to 6 months will be reported as secondary endpoints. These will also include the composite of worsening HF or cardiovascular death and its individual components. Worsening HF was defined as an episode of HF that requires unplanned medical attention with an increase in diuretic dose or intravenous diuretic therapy or HF hospitalization. The total number of HF hospitalizations will be reported. Clinical parameters, including changes in functional NYHA class, NT‐proBNP level, quality of life, and 6MWT distance, will be assessed after 6 months. Additional secondary endpoints are safety endpoints related to procedure success and adverse events. A detailed list of pre‐specified study endpoints is provided in *Table* *2*.

**Table 2:** Primary and secondary endpoints of the study

Outcomes	Time frame
Primary outcome measure	
Change in left ventricular ejection fraction	Baseline and 6 M
Secondary outcome measures	
Time to the first occurrence of worsening HF or cardiovascular death	At least 24 M
Time to the first occurrence of worsening HF	At least 24 M
Time to cardiovascular death	At least 24 M
Total number of HF hospitalizations	At least 24 M
Change in indexed LV end‐diastolic and end‐systolic volumes	Baseline and 6 M
Improvement in clinical parameters (NYHA class, EHRA‐AF, KCCQ score)	Baseline and 6 M
Change in the 6‐min walk test	Baseline and 6 M
Laboratory parameters – NT‐proBNP	Baseline and 6 M
Procedural‐related characteristics (total procedure and fluoroscopy time)	Peri‐procedural
Procedure‐associated adverse events	30 days after the procedure
Need for procedural reintervention	At least 24 M
ECG parameters (QRS duration)	Baseline and after the procedure
Pacing parameters (capture threshold, lead impedance)	At least 24 M
Number of detected sustained VT/VF	At least 24 M

ECG, electrocardiogram; EHRA‐AF, European Heart Rhythm Association score of atrial fibrillation; HF, heart failure; KCCQ, Kansas City Cardiomyopathy Questionnaire; LV, left ventricular; M, months; NT‐proBNP, N‐terminal pro‐B‐type natriuretic peptide; NYHA, New York Heart Association; VF, ventricular fibrillation; VT, ventricular tachycardia.

### Study flow

After enrolment, both interventions will preferably be performed during the same hospitalization. Patients will be followed for a minimum of 24 months after randomization. Regular outpatient visits will be performed at 1, 6, 12, and every 6 months thereafter. Patients will be followed up for the occurrence of the primary and secondary endpoints, echocardiographic examination, and device interrogations. Symptoms will be evaluated with NYHA and EHRA‐AF scores, while quality of life will be assessed using the KCCQ. Data regarding HF hospitalizations and cardiovascular deaths will be collected continuously as presented in *Table* *3*.

**Table 3:** Time and events table of the CONDUCT‐AF trial

	Enrolment (−7 to −1 days)	Implant (day 0)	Discharge	FU 1 (1 M ± 7 days)	FU 2 (6 M ± 10 days)	FU 3 (12 M ± 15 days)	FU 4 (18 M ± 30 days)	FU 5 (24 M ± 30 days)
Eligibility check	x							
Informed consent	x							
Demographics	x							
Comorbidities	x							
Medication	x			x	x	x	x	x
Randomization	x							
Device implantation and AVNA		x						
ECHO	x		x		x			
12‐lead ECG	x		x	x	x	x	x	x
EHRA‐AF score	x				x			
NYHA class	x				x			
KCCQ score	x				x			
6MWT	x				x			
Lab testing	x				x			
Review clinical outcome events^a^				x	x	x	x	x
Device interrogation				x	x	x	x	x

6MWT, 6‐min walk test; AVNA, atrioventricular node ablation; ECG, electrocardiogram; ECHO, echocardiographic examination; EHRA‐AF, European Heart Rhythm Association score of atrial fibrillation; FU, follow‐up; KCCQ, Kansas City Cardiomyopathy Questionnaire; M, months; NYHA, New York Heart Association.

^a^ Hospitalization for worsening heart failure, hospitalization for ventricular tachycardia/implantable cardioverter‐defibrillator shock, need for reintervention, death.

### Trial interventions

Device implantation will always precede AVNA. Crossover between pacing modalities will be permitted in cases of unfeasible LBBAP implantation (due to high threshold, failure to penetrate the septum, or unmet criteria for conduction system capture) or failed BVP implantation due to anatomical constraints that preclude the LV lead from being placed in an acceptable position. The additional defibrillator lead will be implanted at the discretion of the implanting physician as indicated in the guidelines.^25^

#### Biventricular pacing

Implantation of the biventricular device will be performed using standard techniques. The RV lead will be positioned in the RV apex or septum, while the LV lead will be delivered to the most appropriate coronary sinus tributary, preferably the posterolateral or lateral vein. Optimal V‐V delay and appropriate LV vector in a multipolar LV lead will be selected after the procedure to achieve the narrowest possible QRS complex.^17^

#### Conduction system pacing

Left bundle branch area pacing will be the preferred pacing technique in patients randomized to the CSP group. The implantation technique has been described elsewhere.^24^ In brief, after localizing the His bundle area (fluoroscopically or determined with intracardiac signal), the LBBAP lead will be positioned approximately 1–1.5 cm distally to the His bundle position in the RV septum. Before screwing the lead into the interventricular septum, the suitable position will be confirmed by fluoroscopic features and adequate paced QRS morphology (‘w’ pattern in lead V1, polarity discordance of leads II and III). Lead progression into the interventricular septum will be monitored using progressive change of paced QRS morphology, fixation beats, local endocardial electrogram, impedance, and current of injury. Confirmation of conduction system capture will be desired.^26^ Given that the pacing parameters with LBBAP are typically low and stable, a backup RV lead will not be mandatory but can be implanted at the discretion of the operator.

If LBBAP is unobtainable, HBP implantation will be attempted. The procedural steps for delivering HBP were previously reported.^20^, ^22^ In short, the same leads and dedicated delivery sheaths as with LBBAP will be used for HBP. His bundle potential mapping will be performed in a unipolar setting using the electrophysiological system at a sweep speed of 100 mm/s and under fluoroscopic guidance. Distal His bundle potential with large ventricular signal and small atrial signal (ventricular to atrial electrogram ratio >3:1) will be targeted before the pacing lead will be screwed into position. An acute HBP threshold ≤2 V at 1 ms will be considered acceptable. The additional backup lead will be mandatory for all patients receiving HBP devices.

#### Atrioventricular node ablation

Atrioventricular node ablation will be performed following pacemaker implantation (preferably during the same hospitalization), which will temporarily be set to 40 bpm for the duration of the procedure. After femoral vein access is obtained, the ablation catheter will be positioned to the presumed area of the atrioventricular node in the mid‐septum under fluoroscopy and according to the intracardiac electrograms. Ablation will be performed in a temperature‐controlled (40 W, non‐irrigated tip catheter, up to 60 s) or power‐controlled mode (40 W, irrigated tip catheter, up to 60 s). AVNA will be considered successful after clearly documented presence of a complete atrioventricular block that will persist after a mandatory 20‐min waiting period. Each pacemaker will be tested after the procedure and at every follow‐up at a lower pacing rate (30 bpm) to check for any relevant escape ventricular rhythm. Any acute change in device lead position or pacing threshold following AVNA will be monitored. After AVNA, the pacemaker will be initially programmed to operate in VVI mode at 90 bpm base rate. At 1‐month follow‐up, the base rate will be decreased to 70 bpm.

### Echocardiographic examination

An echocardiographic assessment will be obtained before enrolment, after AVNA, and at the 6‐month follow‐up visit. All participating centres will receive a detailed echocardiographic protocol. Transthoracic echocardiographic images will be obtained according to guidelines following the European Association of Cardiovascular Imaging (EACVI) recommendations.^27^ Participating sites will follow a standardized imaging protocol, including specific image acquisition views in two‐dimensional imaging, to ensure uniformity across all study participants. All videos should include five cardiac cycles. During echocardiographic examination heart rhythm and heart rate will be continuously monitored and recorded. As stated previously, at the 1‐month follow‐up, the base rate will be decreased to 70 bpm, and the echocardiographic examination at the 6‐month follow‐up will be performed at the uniform base rate. Images will be acquired using commercially available ultrasound systems and stored in Digital Imaging and Communications in Medicine (DICOM) format. All echocardiographic studies will be anonymized and securely transferred to the independent Core Laboratory (University Medical Centre Maribor, Maribor, Slovenia), which is blinded to treatment allocation. The core laboratory will provide standardized echocardiographic assessments, ensuring consistency in measurement and interpretation. Measurements of LV volumes and LVEF will be performed using Simpson's method of disk summation. To reduce inter‐cycle variability, relevant echocardiographic measurements will be averaged over five subsequent cardiac cycles.

### Study timeline

Recruitment began in July 2023 and is expected to be completed by the end of 2025. Follow‐up is expected to be concluded by the end of 2027. The expected total study duration will be approximately 4.5 years, composed of 2.5 years of patient enrolment and 2 years of study follow‐up. The short‐term analysis of the primary outcome is expected in 2026, while secondary outcomes will be reported by the end of 2027.

### Sample size and statistical considerations

The study is designed to test the two‐sided hypothesis, at a type I error level of 0.05, that participants assigned to the CSP group would achieve a non‐inferior improvement of LVEF compared to the participants assigned to the BVP group, with the non‐inferiority margin of 5%. Additionally, an allowable absolute difference of 5% in echo measurement of LVEF with Simpson biplane was set to cover the intraobserver variability. To estimate the sample size and study power, we used the linear mixed model. Although there is an absence of robust estimation from the literature, according to the non‐randomized study, the predicted mean change of LVEF in the population would be +12% in the CSP group and −1% in the BVP group.^24^ According to available data, the maximum standard deviation of measured LVEF in the CSP group was 13% and 15% in the BVP group.^21^, ^23^, ^24^ Therefore, the LVEF change in the CSP group would be distributed in the population as *N* (12, 13^2^) and in the BVP group as *N* (−1, 15^2^). Based on these assumptions, we calculated that the necessary sample size would be 70 participants (35 patients in each group) to achieve a power of 0.9996 and yield a statistically significant result. The dropout rate was assumed at 15% due to study withdrawal or loss to follow‐up. Consequently, the target patient number was set as 82 participants.

Acknowledging limited data available in the literature, we conducted several sample size simulations to address the robustness of our assumptions across a plausible range of ejection fraction responses after both pacing modalities. Even in a more conservative scenario where the CSP group follows a *N* (7, 13^2^) and BVP group a *N* (0, 15^2^) distribution, the calculated power remains above 80%. The ALTERNATIVE‐AF trial showed improvement in LVEF in both pacing modalities with only 5% absolute difference in favour of CSP.^28^ Even in this scenario (CSP mean ejection fraction change: +12%, BVP mean ejection fraction change: +7%), the calculated power would remain >95% to demonstrate the non‐inferiority.

Data management will be performed using the Research Electronic Data Capture – REDCap (Vanderbilt, Nashville, TN, USA) and statistical analyses using the R programming language. The trial will follow an intention‐to‐treat analysis approach.

### Adverse events

All adverse events and complications will be documented and reported. Interim safety analyses will be performed during the study. Early termination of the study will be permitted if BVP efficacy is superior to CSP or if significant harmful effects are documented with an implanted CSP over BVP.

## Discussion

To the best of our knowledge, CONDUCT‐AF will be the first multicentre, randomized trial to evaluate the efficacy and safety of CSP compared to BVP in patients with refractory symptomatic AF, HF with LVEF <50%, and narrow QRS after AVNA. To date, the only randomized, crossover study, ALTERNATIVE‐AF, comparing BVP and HBP, demonstrated small but significant post‐AVNA improvements in LVEF in a similar patient cohort, but with no conclusive data about clinical endpoints.^28^ The APAF‐CRT study reported a significant reduction in all‐cause mortality, HF hospitalizations, and worsening HF in patients treated with AVNA and BVP.^17^, ^18^ These results further established the potential of the pace and ablate strategy in conjunction with BVP. However, the need for precise tools and implant expertise, a complex device with additional lead and subsequent increased costs, and a longer procedure duration affected by anatomical variations of the coronary sinus limit its wider adoption in clinical practice. On the contrary, CSP might bring several advantages with high implant success rates, stable pacing parameters, and better cost‐effectiveness.^21^, ^29^ Therefore, we can reasonably assume that the positive results of the study might not only confirm CSP as a feasible alternative to BVP but will also further establish the pace and ablate strategy as an important therapeutic option in refractory AF treatment with HF when sinus rhythm is no longer pursued.

Among CSP modalities, LBBAP may be advantageous over HBP due to better pacing parameters and success rates.^21^ Therefore, adoption in clinical practice is attractive despite the lack of data from randomized trials and concerns regarding physiological activation compared to HBP. Namely, LBBAP is achieved by penetrating the interventricular septum to capture the left bundle branch and maintaining near‐normal LV electrical activation with supposedly indirect RV recruitment.^30^ These concerns may not be relevant as propensity score analysis matching 99 LBBAP with 86 HBP patients demonstrated comparable clinical benefits (echocardiographic and HF outcomes), higher implant success rate, better pacing parameters, and fewer complications in the LBBAP group compared with HBP.^31^ Therefore, LBBAP is a promising alternative to BVP that may foster the benefits of the pace and ablate strategy. Consequently, a randomized clinical trial comparing the outcomes of CSP and BVP in patients with HF and refractory symptomatic AF undergoing subsequent AVNA is of interest.

To conclude, the CONDUCT‐AF study directly addresses clinical decision‐making on optimal pacing modality in HF patients undergoing AVNA, potentially providing the data that will impact future guideline recommendations.

### Study limitations

A potential limitation of this study is the small sample size that was calculated to achieve statistically significant results and prove the non‐inferiority of CSP compared to BVP in the primary outcome – change in LVEF. Although our study may provide some insight into hard endpoints, adequately powered, large‐scale randomized clinical trials with long‐term follow‐up will be required to confirm our findings regarding morbidity and mortality. As only patients with reduced LVEF and narrow QRS will be included, the results cannot be extrapolated to other subgroups of patients undergoing AVNA.

### Funding

The CONDUCT‐AF trial is an investigator‐initiated, independent clinical trial. This work was partially supported by an institutional (UMC Ljubljana) research grant [TP 20220047]. No additional grants from funding agencies in the public, commercial, or non‐profit sectors were obtained.

**Conflict of interest**: A.Z.M. reports receiving proctoring fees from Abbott. M.J. reports proctorship fees from Abbott and Johnson & Johnson. C.P. reports receiving travel and proctoring fees from Medtronic and Biotronik. S.B. reports receiving speaking honoraria, travel, and proctoring fees from Medtronic, Biotronik, and Abbott. P.B. reports receiving travel and proctoring fees from Biotronik. V.T. reports receiving speaking honoraria, travel grant, and proctoring fees from Medtronic, Abbott, and Biotronik. D.Ž. reports receiving proctoring fees and speaker honoraria from Medtronic, Biotronik, Abbott, and Boston Scientific. All other authors have nothing to disclose.
